# Nanosystems, Edge Computing, and the Next Generation Computing Systems

**DOI:** 10.3390/s19184048

**Published:** 2019-09-19

**Authors:** Ali Passian, Neena Imam

**Affiliations:** Computing & Computational Sciences Directorate, Oak Ridge National Laboratory, Oak Ridge, TN 37830, USA; imamn@ornl.gov

**Keywords:** edge computing, the internet-of-things, carbon nanotubes, processors, nanoscience, quantum computing, neuromorphic computing, plasmonics, photonics, information technology

## Abstract

It is widely recognized that nanoscience and nanotechnology and their subfields, such as nanophotonics, nanoelectronics, and nanomechanics, have had a tremendous impact on recent advances in sensing, imaging, and communication, with notable developments, including novel transistors and processor architectures. For example, in addition to being supremely fast, optical and photonic components and devices are capable of operating across multiple orders of magnitude length, power, and spectral scales, encompassing the range from macroscopic device sizes and kW energies to atomic domains and single-photon energies. The extreme versatility of the associated electromagnetic phenomena and applications, both classical and quantum, are therefore highly appealing to the rapidly evolving computing and communication realms, where innovations in both hardware and software are necessary to meet the growing speed and memory requirements. Development of all-optical components, photonic chips, interconnects, and processors will bring the speed of light, photon coherence properties, field confinement and enhancement, information-carrying capacity, and the broad spectrum of light into the high-performance computing, the internet of things, and industries related to cloud, fog, and recently edge computing. Conversely, owing to their extraordinary properties, 0D, 1D, and 2D materials are being explored as a physical basis for the next generation of logic components and processors. Carbon nanotubes, for example, have been recently used to create a new processor beyond proof of principle. These developments, in conjunction with neuromorphic and quantum computing, are envisioned to maintain the growth of computing power beyond the projected plateau for silicon technology. We survey the qualitative figures of merit of technologies of current interest for the next generation computing with an emphasis on edge computing.

## 1. Introduction 

Capturing and isolating single atoms or molecules and controlling their quantum states to achieve the desired function is undoubtedly a fantastic milestone for toolmaking and information processing, the two pillars of human endeavors. Stimulated by successful demonstrations, such as trapping a single atom or imaging single atomic sites and bonds within a molecule, there have been visions to reach beyond nanotechnology for continuing at thousand times smaller into the pico-technology [1], and at a million times smaller into the femto-technology [2], the realm of neutrons, protons, electrons, and other nuclear particles. Amazingly, pico- and femto-technologies are already being contemplated for addressing technology bottlenecks, such as a better electronic on-off switching speed to improve communication bandwidths beyond ~50 GHz [3]. In the earlier stages of nanotechnology, in “Technical boundless optimism”, D. Jones, reviewing “Nano! Remaking the World Atom by Atom”, raised certain skepticism with respect to the promises and potentials of nanotechnology [4]. However, demonstrations, such as resolving the atoms that make up the benzene rings within a molecule (pentacene) adsorbed on a copper or a sodium chloride surface [5], uphold the noted optimism. Resolving the atomic structure of graphite, monitoring the formation of fullerene C_60_ molecules [6], and discerning the various carbon-carbon bonds within the molecules [7] are other examples of boundless success. In “Bonding more atoms together for a single molecule computer”, C. Joachim discussed how the future of computing would depart from solid-state integrated electronics and enter the realm of molecular transistors [8]. Such claims are already being supported by works on single molecules, for example, controlling cis-trans transition in Azobenzene molecule, leading to the molecules being “switched” with spatial selectivity, has been demonstrated [9]. Taming individual atoms towards quantum computing, atom-by-atom assemblers to arrange several trapped neutral atoms in one-dimension [10], in arbitrary two-dimensional patterns [11], and in three-dimensional arrays [12] with controllable single atom capability, have been demonstrated. To scale up the “fabrication” of such atomic and molecular switches, novel concepts are being reported, including the demonstration of monolayer surface patterning at 3.5 nm on a gold surface via self-assembly, offering a potential path to large-area patterning [13]. 

Assessing the joint impact of nanosystems that function as sensors, actuators, processors, memory, and communication links is nontrivial. For example, the importance of electronic computers in investigations of molecular quantum mechanics was recognized as early as 1956 [14]. However, the notion of molecules themselves being used as computers, or quantum effects being employed to compute and communicate, have only emerged recently. Astonishingly, DNA computing [15], molecular machines [16], biological microprocessors [17], bio-electronic computers [18], etc. have been already reported, albeit largely exploratory.

Nevertheless, aided by nanosystems, information is being created at rapidly increasing rates. Reciprocally, the explosive growth of data [19] and its profitable global market [20] is rapidly advancing the exploration and discovery of new nanosystems that can statically and dynamically accommodate information [21]. The generation and fate of information and its fascinating dynamic relationship with information technological devices warrant scrutiny [22,23,24,25,26,27]. Morphing into a countless number of sensors [28,29,30], data collectors are generating mindboggling amounts of data [31], soon to reach ~10^21^ bytes (or zettabytes, ZB) [32]. A 2012 industrial study reported an estimated 1 ZB of data generated worldwide with a predicted 40 ZB by the year 2020 [33]. In year 2011, Hilbert and López, estimating the world’s technological capacity to store, communicate, and compute information, concluded that in the year 2007, the world had stored ~0.29 ZB (compressed bytes), communicated ~2 ZB, and carried out 6.4 exaflops (=6.4 × 10^18^ flops or floating-point operations/s) [19]. For comparison, Hilbert and López noted that the exaflop rate roughly equals the maximum number of nerve impulses/s executed by one human brain, and the ZB stored data is approaching the roughly 100 ZB stored in the DNA of a human adult [19].

With computing operation rates at the exaflop in the horizon [34], the high-performance computing (HPC) has to possess the capability to handle exabyte (~10^18^ bytes) massive quantities of data in addition to improved flops. The processing, communication, and storage of the large volumes of data by transistor circuits, interconnects, and networks, invented to make use of the digitally represented information, are pervasive. However, these operations are growing increasingly challenging due to data traffic, memory, and computing capacities. To combat the challenges associated with the need to transfer and communicate large amounts of data generated in one location to an HPC data center in a different location, the concept of edge computing (EC) [35] is being intensively investigated [36,37,38,39]. 

**Figure 1:**
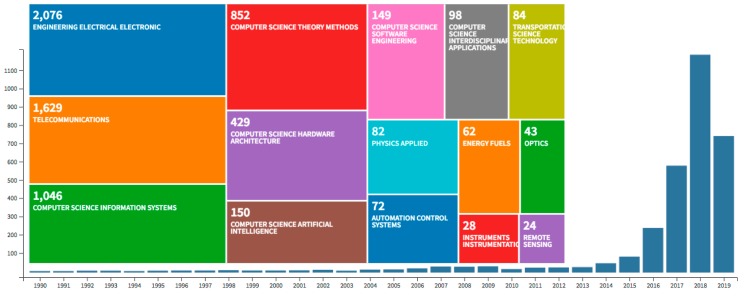
Article statistics showing the histogram of edge computing over publication years, a compilation from [54]. Inset: distribution by discipline [54]. Notable are the intensified research, the multidisciplinary character of the field, and the largest contributing disciplines. The number of other contributing disciplines (not shown) is also increasing rapidly.

The intent of this article was a survey of the general state of the EC and the pertinent nanoscience subfields, including nanophotonics [40,41,42,43,44,45] and nanomaterial-based components [46,47]. Noting the cross-disciplinary nature of the solutions needed to overcome existing challenges in EC, we discussed how the relevant research areas and technologies could be mutually beneficial towards serving the needs of the internet of things (IoT) and HPC in the exascale regime. A growing number of exploratory work of potential for the next generation computing is being reported, e.g., creating and controlling Majorana quasiparticles for use in quantum computing [48], or use of metamaterials in optical computing of integral equations [49]. However, while several exciting venues have opened up towards achieving stable information carriers for quantum computing or biologically inspired massive processing, we here emphasize developments that have been recognized to be closer to scalable implementation. Skyrmions, for example, are spatially highly localized (~nm) excitations or quasiparticles in a magnetic material. They exhibit a level of stability, mobility, and localization that render them highly suitable for information encoding. These magnetic spin configurations are protected by the specific topology of the underlying material domain (e.g., a ferromagnet-heavy metal bilayer [50]). Thus, they can be transported and manipulated to convey information, leading to the notion of Skyrmionics, which has been proposed for stochastic or probabilistic computing [51]. However, probabilistic computing (compared to binary-encoded computation) is itself under active exploration to potentially provide an alternative computing platform for embedded systems at lower area and power, and better error resilience, and high computational density, all of which are ideal attributes for constrained environments, such as EC devices (sensor nodes and mobile devices) [51,52,53]. Skyrmionics, however, is not considered sufficiently developed to compete with existing silicon electronics. Figure 1 displays some elementary statistics related to EC, emphasizing the recent rapid growth and the diversity of EC research profile. We noted that the key discussion of the article, that is, the use of nanotechnology in the development of EC, pertains more generally to the next generation computing systems rather than specifically to EC. 

## 2. Edge Computing

**Figure 2:**
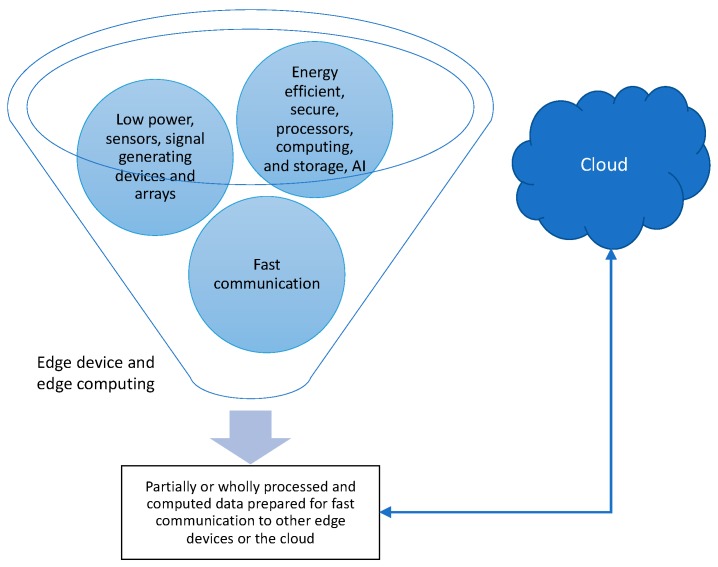
Some basic elements of a device in the edge computing paradigm. The edge device does not necessarily require a connection with a centralized cloud. Many challenges lie ahead regarding energy efficiency, data quality and reliability, data and device security, computing performance level, etc., stimulating exploration for novel nanosystems and processor architectures, rapid communication, and related components.

Edge computing may be regarded as a product of the evolution of electronics and communication. In “How we created edge computing” [35], M. Satyanarayanan discussed how the realization of the limitations of cloud-based processing led to the proposal of the concept of EC via the introduction of cloudlets [55,56,57]. While sharing similarities, clear distinctions exist between cloud, cloudlet, fog, and edge [37,38,39]. Satyanarayanan et al. subsequently showed that reductions of 51% and 42% in latency and power use, respectively, could be achieved in a mobile device when using cloudlets (in this case, a virtual machine on Dell Optiplex 9010) instead of the cloud (Amazon EC2). In “Working on the edge”, further definition of EC was provided by V. Bahl, who also discussed the future of EC, and how it would help the cloudification of the telecom network and become an integral component of it. Interestingly, Bahl further characterized the EC as a “marriage” between the telecom and the IT industries [58]. While the definition of EC may be subject to slight variation and remains largely broad, for the sake of our presentation, we showed the common ingredients in Figure 2. 

Examining the rapidly growing number of investigations reported (see Figure 1), the following recurring definition takes the center stage: EC is an emerging data processing paradigm toward countering the current and projected bottlenecks of cloud-based computing. Thus, EC strives to complement the cloud. An EC device processes data on local computing and communication infrastructure and only, if necessary, prepares data and establishes a communication link to a data center or other EC devices. Therefore, EC is envisioned to overcome both latency and memory bottlenecks of the current centralized cloud-based paradigm. However, despite recent related surveys [37,38,39], exactly how an EC device should be defined has not been rigidly formulated due to the infancy of the EC field and the high degree of diversity of IoT. Nevertheless, we may recognize that to constitute an EC device, innovations in both hardware and software are necessary to meet the growing speed and memory requirements. 

EC devices take on the task of carrying out preliminary data processing instead of transmitting raw data to data centers for processing. Aiding the cloud by greatly reducing the upload bandwidth and computation complexity, the EC nodes are envisioned to perform tasks, such as real-time signal and image processing, combinatorial optimization, agent-based modeling, big data analysis, etc. Such tasks are performed to provide secured services, effective control, and seamless decision-making while achieving energy efficiency. Therefore, HPC is essential to EC networks [59]. In recognition of the importance of the EC field, in, a recent, editorial “Take it to the edge” [60], it was named the 2019 technology of the year. 

**Figure 3:**
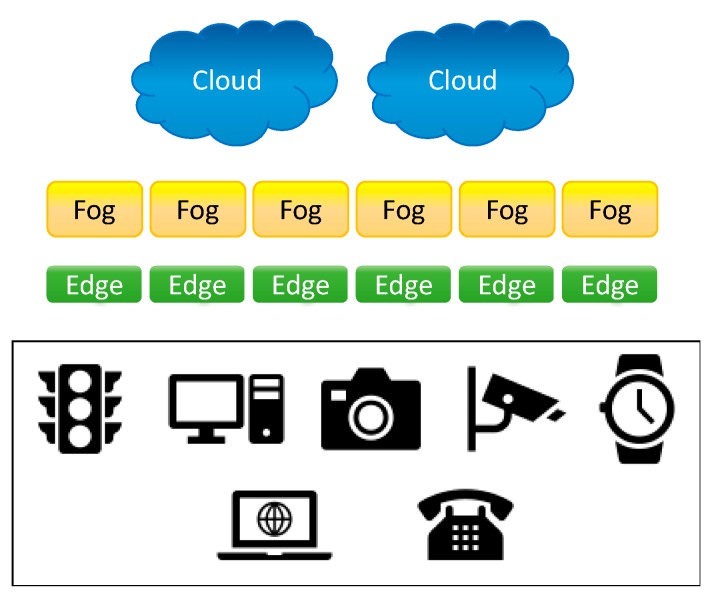
Multi-tier computing networks. The integration of various technologies to achieve intelligent applications and services.

A central objective of cloud, fog, mist [61], and recently EC, has been to achieve enhanced performance locally using a non-centralized distribution of computer memory and computing power (see). The advantage of offloading computational tasks to fog or cloud servers is a time reduction for task processing. Thus, physically, servers are deployed at the near edge or the extreme edge of the network (closer proximity to the data sources) instead of the data centers [62]. Specifically, EC is addressing the challenges facing the computing of data for which speed and scale are not only ideal but necessary. Despite the power of the cloud computing and storage solutions (e.g., platforms, such as Amazon elastic compute cloud [63] and Google cloud platform [64]), the increasing number, type, and spatial distribution of devices, generating extremely large amounts and types of data, require continuous innovation. Therefore, the infrastructure is evolving from its core, i.e., the data centers to its edges. The diversity in the IoT applications implies that data processing may require different levels of intelligence, efficiency, and security. As the technologies could generate predictions about personal behaviors and private lives, measures must be considered to guard against the associated pervasive analytics. An important issue of EC is dealing with data protection, as discussed in “Data protection in the age of big data” [65]. Recently, in “Multi-tier computing networks for intelligent IoT”, as depicted in Figure 3, Y. Yang defined the roles of cloud, fog, and edge computing technologies; delineated their hierarchies; and described how integration among them might be necessary for optimum IoT services [66].

**Figure 4:**
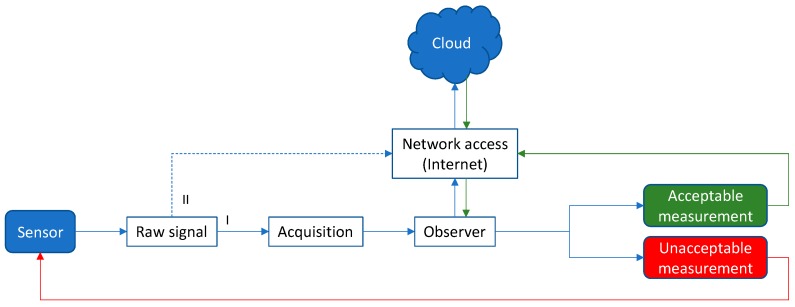
Example of a current paradigm based on cloud computing. Sensors generate raw signals, which are submitted to the cloud directly (route II) or are acquired and observed and then either communicated to the cloud or are evaluated and submitted to the cloud.

Increasingly advanced sensors with sophisticated data acquisition (software and hardware) are being employed globally. In sensing, a simple use case is that of a single sensor or a network of complex integrated sensors [67] reporting a single or arrays of parameters, e.g., extracted from a terrestrial or an extraterrestrial environment. As an example, consider a sensor for the detection of environmental methane, mercury, fungi, or bacteria. As depicted in Figure 4, a raw signal is produced by the sensor, which within some calibration is representative of or proportional to the presence of the sought chemical or biological species. To create useful information and decision-making, the raw signal is either (I) communicated directly to the cloud via a network for processing and storage, or (II) is first somewhat processed locally and then communicated to the cloud. 

**Figure 5:**
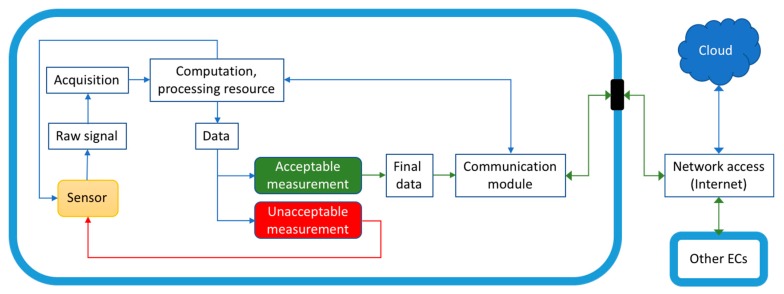
A simplified edge computing approach. A sensor generates raw data, which is locally processed and evaluated. If the outcome meets certain criteria, the data is then communicated to the cloud for further processing/computing and storage.

Within the IoT, the number of sensors is exploding, and thus feeding or uploading such raw or insufficiently processed varying sized signals is readily seen to lead to bandwidth, latency, and storage problems [68]. For example, smart cities [69], homes, and cars will generate a heretofore unimaginable amount of data and communication loads. EC is envisioned to alleviate such predicted/expected problems. By incorporating a local high-performance processor with built-in artificial intelligence (AI), local decision-making can be carried out and only if necessary, communicated with the cloud. This is depicted in Figure 5, where the raw data is locally processed in an embedded processor with sufficiently provided AI (e.g., Fuzzy logic, Bayesian network) to generate high-level information and confident decisions. The output information, instead of the raw data, may then be communicated to the cloud for further processing (HPC, storage, etc.).

Distributed sensing, such as used in the oil and gas industry, is expected to be a major beneficiary of EC. For instance, spatially distributed, fiber optic sensors, capitalizing on Brillouin and Raman scattering [70], interact and detect pressure, vibrations, gas species (leaks and contamination), temperature, and other harsh-environment parameters for fossil energy research [71]. Due to its distributed nature, EC encompasses many of the same aspects of signals, processing, computing, and data storage. As a result, within the IoT, including IIoT (industrial IoT [72]) and IoMT (internet of medical things [73,74]), the EC devices are extremely diverse, and the volume of data they are generating and processing is rapidly increasing. Data formats include time and frequency space signals, complex images, sound and voice, and a plethora of protected health [74,75,76], personal, and sensitive data. Due to the variety of EC devices, data types, and algorithms [77], many AI-based or smart offloading and transmission strategies are being proposed, such as employing machine and deep learning methods [75,78], or mimicking human brain networks [79]. Similarly, knowledge-sharing strategies to take advantage of self-taught knowledge between EC devices, such as pertaining to home IoT, have been considered [80]. Undoubtedly, EC-specialized or related optimization problems formulation and solution will be valuable [72,81,82,83], as discussed in the case of optimization consideration for platooning for automatic driving [84], evolutionary game theoretical proposals for mobile devices and security [85,86], better estimation of interference in EC devices [87], cost-effective placement of EC servers [88], computational power allocation for blockchains [89], and incorporation of computer vision [90]. 

While security strategies are being developed [61,91,92], the emerging era of EC offers tremendous opportunities for research and development in nanosystems, such as optical sensors, optical communication, and photonic processors [40,41,42,44,93,94], all of which are to be seamlessly integrated in one or more part of the secure network, from the edge to the core. 

The increased computational loads on end EC-based devices, in conjunction with IoT operating system, is to consider balanced process management for the interplay between the processing and communication tasks [95] or resource and energy consumption [96]. In an empirical study using IoT sensor devices, tests were carried out with different levels of computational load and various priority schemes to show that an increased load results in cross-effects between the processing and communication tasks, significantly affecting their performance [95].

The decentralized decision-making of the EC paradigm toward generating accurate data must be energy efficient. For example, the continuous readings to provide high-resolution location can be energy inefficient in mobile devices prompting proposals to develop on-device cognitive-inspired control for power-aware human mobility analysis in IoT devices [97]. In addition to energy efficiency, the emerging EC, aiming to provide faster IoT operation and mobile devices, seeks to conserve and optimize memory, cache, server placement, size, weight, etc., calling for new studies and proposals. Recently, to realize a potentially scalable and intelligent caching scheme that aims to reduce cache redundancy, a progressive popularity-aware caching scheme was proposed [98]. To avoid wasting cache space when the content is not popular enough, the proposed method first caches initial “chunks” of the content at the edge node and then progressively continues caching subsequent chunks at upstream according to the content popularity and each content node position [98]. Also, the cost-effective placement of edge servers has been proposed for metropolitan area network [88]. 

The universality of EC applications and the accumulative impact they will impart can be readily appreciated from the long list of applications that are rapidly growing into various hierarchies of service. For example, for more reliable service, solar cells/panels and related devices need to become smart with AI for IoT in an EC domain. Spectral variation, combined with an array of environmental sensors to monitor, pressure, temperature, humidity, wind speed, cloud movements, etc., can be utilized to optimize the performance of solar cells, leading to potentially significant economic benefits. Similarly, traffic lights augmented with hyperspectral imaging and chem-bio sensors can be made locally smart when combined with weather sensors and the unique local population and infrastructure signatures. More concrete EC applications include scalable framework for early fire detection [99], disaster management services [100], accelerometers for structural health monitoring [101], micro-seismic monitoring platform for hydraulic fracture [102], a framework for searchable personal health records [75,76,103], smart health monitoring [76,104] and healthcare framework [105], improved multimedia traffic [106], a field-programmable gate array (FPGA)-based system for cyber-physical systems [107] and for space applications [108], biomedical wearables for IoMT [73,76,109], air pollution monitoring systems [110], precision agriculture [111,112], diabetes [74] and ECG [109] devices, and marine sensor networks [113]. 

Acute needs of edge devices are readily identified within the customs and border protection (CBP), where agents controlling illicit drugs and contraband can immediately make decisions instead of communicating with other data centers. With the opioid crisis, the number of sensors will inevitably increase, and EC devices with sufficient computing power and rapid communication rate can provide critical decision-making. 

Since the EC devices, such as IoT sensor nodes, will be ubiquitous, embedded computing paradigms for EC devices will have to use energy-efficient microprocessors to process the data. Furthermore, supplying real-time on-demand energy for EC devices will have to be investigated, in particular, since the energy consumption rate changes dynamically at different nodes prompting new charging scheduling schemes [114].

For chemical and biological sensors, real-time data acquisition, rapid processing, and computing are necessary. If the sensor output can be processed locally, instead of being sent to a different location, better mitigation and remediation can be achieved. A closely related EC-use case is the need for intelligent surveillance cameras. With the tremendous need for chem-bio standoff detection, the ability to collect molecular spectral data in addition to visual information would revolutionize the surveillance technologies. Aided with AI, EC surveillance cameras will be capable of processing the local streaming and only communicate specific detection results rather than continuous submission of the data. Upon reception, the cloud could then send the EC device new instructions, including programming to different functionalities. 

Increasingly more complex services are expected to be provided by the continuously improving energy efficiency of system-on-chip (SoC), furnishing sensors and devices (edge, mist, fog, and IoT end devices) with significant computing power. This allows a self-contained module in which edge sensors and actuators generate data that can be processed on-site. In addition to software, SoCs, containing components, such as processors, graphics processing unit GPU), network-on-chip (NoC) [115], and memory and data storage, essentially function as a server. Attractive/desired attributes of SoCs include their SWaP (size, weight, and power). 

**Figure 6:**
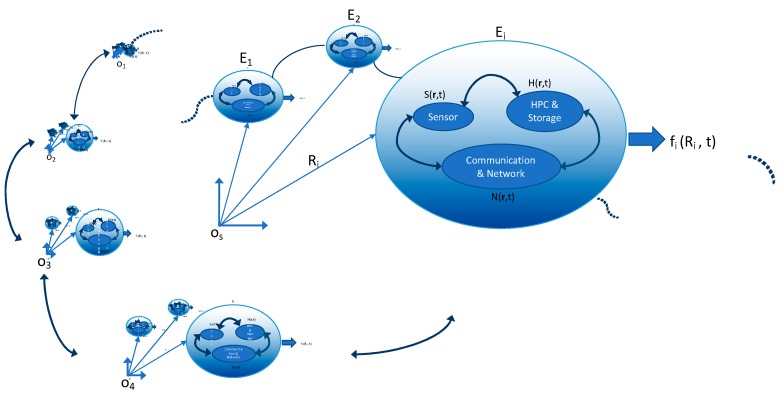
Envisioning a potential variation of the interconnectivity of edge sensors. The nested growth of edge devices may form a system of a coupled dynamical system with fractal self-similarity. The sensor output *S* can be processed to *H* and communicated as *N* with a final output of *f* for node *i* located at *R_i_* relative to data center *O_s_*.

Given the staggering number of future EC devices, it may be prudent to treat certain dynamic aspects of the EC within the realm of coupled oscillator systems and self-similar geometries, as graphically depicted in Figure 6. One may then ask the question of specific distributions of EC devices corresponding to holistic and emergent information phenomena. 

Although there is an increasing number of EC-related optimization work being reported, obtaining quantitative data on the figure of merit of EC is less prevalent in the current state of affairs. Such quantitative data would provide energy consumption merits or other advantages of EC. For example, in a fog versus cloud computing experiment, in which 25% of the applications needed real-time services, around 40% reduction in energy expenditure was reported for the fog [37]. Similar reports are emerging, where also EC advantages are being specifically compared to cloud computing. For example, an edge micro-seismic monitoring system was shown to perform at higher efficiency with less transmitted data when compared to a method lacking edge capability [102]. EC-specific quantitative metrics is currently highly needed. Unlike cloud computing simulation frameworks (including the cloud-based EdgeCloudSim simulator [116]), simulators that could be specifically used for modeling the design and behavior of an EC device are not quite available yet [36].

Given the very broad scope of computing, our review encompassed a brief account on the emerging nanomaterials and nanosystems of potential use in computing. Discussions pertaining to the many types of processors: central processing unit (CPU), GPU, FPGA, etc., architectures (multi-core, heterogeneous core processors, neuromorphic, etc.), and related hardware and software are presented marginally [117,118,119]. 

## 3. Edge Computing Processor Architectures

Although currently, supercomputers, such as Summit and Sierra (ranking No. 1 and No. 2, respectively on the Top500 list) are not practical for EC, it is envisioned that smart supercomputers will eventually enter the EC domain, unleashing new capabilities. Summit [120], for example, a US Dep. of Energy machine at Oak Ridge National Laboratory, links more than 27,000 GPUs and 9000 CPUs to provide 200 petaflops (=2 × 10^17^ operations/s) HPC at a power consumption of 13 MW, which is not practical yet for EC devices. 

The projection that the number of IoT devices will continue to grow rapidly (~50 × 10^9^ by 2020 [37]) places increasing demand on-chip performance. The power efficiency of EC devices is thus of paramount importance despite the development of better energy storage and power transport technologies. Designers, developers, and manufacturers compete to achieve smaller, lighter, lower cost, but faster, higher performance, and more energy-efficient processors for EC applications. Thus, as the EC-use cases are being more systematically characterized, the design of EC-optimized processors is expected to intensify. 

**Figure 7:**
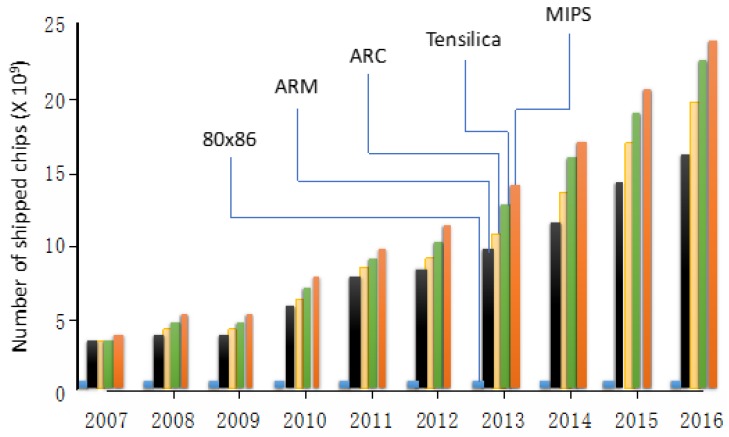
Comparison of the number of RISC chips (ARM, ARC, Tensilica, or MIPS ISAs) versus CISC architecture (Intel’s 80 × 86). RISC, reduced instruction set computer; CISC, complex instruction set computer; ISA, instruction set architecture; ARM, advanced RISC machine.

Processor design begins by considering a specific instruction set architecture (ISA), which provides the needed information to write machine language programs and implement different processors. The ISAs, having varying degrees of complexity [121], can lead to processors that may be more suitable for EC and edge-native applications. For example, an ISA that retains specialized instructions, including those that may be used less frequently in practical programs, is the complex instruction set computer (CISC), which was the basis for Intel’s 80 × 86 chip. CISC, however, has not been seen as competitive for EC, which focuses more on specific performance and functionality criteria. A lighter version of ISA, the reduced instruction set computer (RISC), prioritizing the frequently used instructions while implementing the less frequently used instructions as subroutines, offers a more efficient approach for EC and edge-assisted applications. The use of RISC processors has come to dominate many embedded applications markets making up 99% of microprocessor volume in 2017 [122]. A comparison of chips based on various architectures is shown in Figure 7, where the RISC chips, such as advanced RISC machine (ARM) and MIPS (microprocessor without interlocked pipelined stages), are seen to dominate the market in comparison with Intel’s 80 × 86, which in the year 2011 reached its peak at 0.365 billion [122]. Since RISC processors carry out fewer computer instructions, they operate faster, which is of specific importance in many EC devices where the real-time or simultaneous response is desired. By excluding instructions that are not needed, RISC processors, employing a reduced number of transistors, use a fraction of the power required by CISC processors. RISC processors can also be more suitable for miniaturized EC devices since the size of the semiconducting material (die size) needed for the integrated circuit is proportional to the transistor count, which leads to smaller processors. 

As discussed by D. Patterson in “Reduced Instruction Set Computers Then and Now” [122], RISC grew out of an attempt to execute more instructions in a single short cycle. With the rapidly advancing IoT applications, placing increasing demands on EC, new and innovative ISAs, such as the open-source RISC five (RISC-V) [121,123], is paving the way for new architectures, allowing hardware designers to implement powerful processor for both EC and the cloud [124]. With frozen base instructions (while supporting custom instructions for designing specialty functions), software written for RISC-V will indefinitely run on other similar RISC-V cores. 

Examples of new commercial processors for EC applications include chips from the Advanced RISC Machine (ARM) [125]. In addition to products such as the Cortex families [126], the recently announced Neoverse solutions are explicitly advertised for EC-use cases [127]. The main characteristics that advertise these new chips within the EC domain include low latency, low power consumption, and smaller size. Others offering or competing to supply EC hardware, capabilities, and services include NVIDIA EGX platform [128], APC’s Edge Computing Solutions [129], Open Edge Computing Initiative [130], and others. 

Classically, achieving optimum control is recognized as the hardest part of computer design [121]. However, the impact of novel ISAs stretches beyond classical computing. Interestingly, similar RISC-inspired considerations have also been proposed in quantum computing, to offset any disadvantages that are imposed by the inaccessibility to specific quantum bits (qubits) (e.g., individual trapped ion [131] or individual neutral atom sites [132] in optical lattices) towards implementing control [133]. It may, therefore, be envisioned that similar reduced ISA advantages may prove useful to consider in exploratory nanoscience work on novel processor designs. 

## 4. Nanosystems and Nanoscience: From Edge Sensing to Edge Computing 

Computing has successfully capitalized on the electronic properties of silicon and silicon-based electronics. The success builds primarily upon the scalability of silicon transistors, such as a field-effect transistor (FET), which is the basic component of present computer circuitry. To surpass sub-20 nm nodes, that is, the semiconductor manufacturing process that generates transistors with a size smaller than 20 nm, the conventional scaling has reached major limitations. Currently, silicon FETs are fabricated at the 14-nm node (and at sub-14-nm node using FinFET technology) and have an overall lateral footprint of ~90–100 nm. Innovative approaches and extreme ultraviolet lithography to print the features are being explored towards 5 nm node design. Thus, nanoscale phenomena are expected to be manifested more strongly in the increasingly nanosized domains. In nanosystems, because the electronic and optical response of bulk materials are altered by size, shape, and surface, confinement effects can be pronounced and thus provide new functionalities. The increased surface-to-volume ratio and subwavelength or mean-free-path proportional characteristic length scales and domains are behind many of the observed phenomenal nanoscale effects. For example, a semiconductor material engineered by spatial confinement, creating subbands and thus intersubband transitions, can lead to the generation of radiation (e.g., quantum cascade lasers). In all-dielectric stacks [134,135], similar to semiconductor heterostructures [136], the alternating layers of specific dielectric functions (e.g., small imaginary part, high and low real part dielectrics) create useful photonic band structures [137] or giant (~10^4^) surface [137] or bulk field enhancement [135]. 

In the general case of a composite nanostructured material domain, such as a quantum well [138] of a given morphology or a gap-plasmonic structure [139,140], the ensuing quantum confinement furnishes a variety of enabling mechanisms [141,142,143] via tunneling, modification of local density of states, frustrated total internal reflection, mode coupling, etc. It is envisioned that the opportunities offered by fields, such as advanced nanophotonics [40,42,144,145], nanomaterials [47], and smart sensors, will be capitalized by EC to advance IoT and other network-based applications [113]. Nanosystems, such as nanophotonic crystal cavities, quantum dots, carbon nanotubes (CNT), and nanomaterials, and their composites allow information processing. Owing to the unique properties of nanomaterials and nanostructures, future processors [146] and integrated circuits [147,148] will exploit these nanosystems not only for computing and conveying information but also for storage of information [46,149,150]. 

To address the material challenges associated with the down-scaling of chips, new conductor materials, e.g., graphene nanoribbons [151] to create alternative interconnect [152,153] to replace copper and tungsten are needed [154]. In particular, obtaining new materials, physical basis, and mechanisms of potential to achieve improved processors, circuits, devices, and networks constitute one of the most impactful scientific challenges [155,156,157,158,159,160,161,162,163,164,165]. Searching for novel materials of potential use in HPC, several candidate materials have been proposed, including CNTs [166,167,168,169] and topological material design [170,171,172,173,174,175,176]. Extensive material characterization involving scanning probe microscopy and spectroscopy, and an array of surface probes and other analytical techniques are being employed to acquire a better understanding of nanomaterials. For example, many nanoscale transport properties of interest in transistor or chip design, e.g., plasmon transport in a nanowire or an electron or a phonon transport in a CNT or a semiconducting quantum dot, the mean-free-path (for scattering from impurity or other potentials) can be large leading to the electron or phonon motion being ballistic. Owing to unique light-matter interactions, electronic, mechanical, and thermal properties of nanosystems and innovative approaches to creating superior processors are evolving rapidly [41,46,146,177]. 

**Figure 8:**
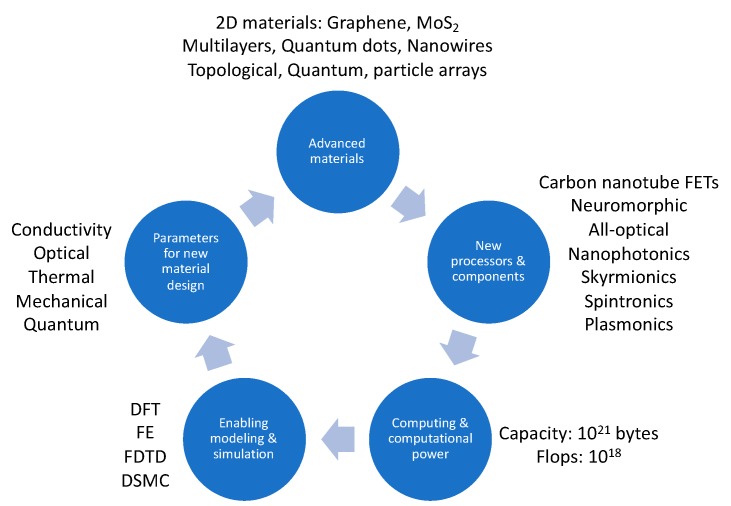
The process loop for the development of new processors. Examples of computational approaches including DFT (density functional theory), finite elements (FE), finite difference time domain (FDTD), and direct simulation Monte Carlo (DSMC) are given only generically.

Since the invention of the point-contact transistor [178], transistors have evolved into the semiconducting building blocks of circuits built onto chips that make up the processor. CMOS (complementary metal-oxide-semiconductor) has been the workhorse of modern digital systems. While the CMOS technology enabled the creation of HPC, new functionalities and types of nanomaterials will enable the evolution of HPC [179,180]. The higher density of information-processing materials that also exhibit superior performance metrics of lower propagation delay and total power dissipation is envisioned to emerge from new nanomaterials, as depicted in the process loop in Figure 8. 

For EC devices, minimizing power dissipation, which is related to the switching mechanisms in various transistor configurations, is critical. For example, switching in tunnel FET (TFET) occurs when barrier tunneling is modulated, whereas, in standard MOSFET (metal–oxide–semiconductor field-effect transistor), one modulates the thermionic emission across the barrier. The MOSFET operates in the subthreshold region when the gate voltage is below the threshold voltage. The subthreshold swing (related to MOSFET’s I-V characteristic) of 60 mV/decade (a decade corresponds to a 10-times increase of the drain current), that is, if the gate voltage is increased by 60 mV, the drain current will increase by a factor of 10. The subthreshold swing 63 mV/decade of drain current at 300 K is a limit imposed by the charge carriers obeying the thermal Maxwell–Boltzmann distribution. Lower supply voltage transistors lead to the lower power consumption of the device. However, in FETs, the thermionic limit (~60 mV/decade) restricts the voltage. Using a graphene source with a CNT channel, a lower voltage of 40 mV/decade has been demonstrated [181]. Similarly, the lower voltage has been achieved in vertical gate-all-around nanowire GaSb/InAs (gallium antimonide/indium arsenide) TFETs, where a subthreshold swing of 40 mV/decade and a current of ~40 μA/wire have been reported [182], or in the band, structure engineered GeSn (Germanium-Tin) TFET [183]. 

The prime example of 1D and 2D nanomaterials of current interest within the enormous application space, comprising next-generation computing systems and circuit elements, includes CNTs and graphene, MoS_2_, transition metal dichalcogenides, black phosphorus [139,184,185], etc. Building processors based on carbon nanomaterial FETs (MOSFET, CMOS, and the multi-gate transistors FinFET [186,187]) have been already demonstrated as in the case of CNT FET. Advanced FETs, such as CNT-based FinFETs [188,189] are being explored as a power-efficient node-scaled platform towards a chip with reduced transistor dimensions and thus increased density. Use of other nanomaterials of relevance includes fabrication and testing via self-assembly of block copolymers to achieve 7 nm node FinFETs [187], and Si, Ge, and SiGe nanowire FinFETs [190]. Block copolymers, belonging to the class of linear copolymers in which the different types of monomers cluster together to form blocks of repeating units, can controllably self-assemble at the nanoscale to form an important class of nanocomposites [185,191]. The resulting functional nanoscale objects can be conductive and semiconductive and, therefore, important to the electronics industry.

Other uses of polymeric nanomaterials include shape memory functionality, explored by IBM (international business machines) corporation through the “millipede” system aimed at developing ultrahigh density storage devices with terabit capacity, small form factor, and high data rate [192,193]. The thermomechanical data storage [193], based on the nanoscale heat-induced deformation in polymers to record and erase information to create novel high-density storage [192], has been explored using the capabilities of scanning probe microscopy (SPM), more specifically AFM (atomic force microscope). In addition to having been employed in the characterization of a plethora of solid-state electronics and photonics systems at the nanoscale, AFM is critically used in exploring the emerging high-density storage technologies beyond hard disk drives and flash memory [194]. Another instance, in which a probe-based approach is indispensable, is the use of ferroelectrics as a memory medium. In ferroelectric memory (capacitor type), the spontaneous polarization state of the crystal domains can be used to store bits of information, which can be changed via an external field [194]. For high-density data storage and rewritable data recording, the thin (few crystal lattice parameters) domain walls in ferroelectrics is a potential advantage since polarization changes occur over short distances.

The evolution of computer technologies, enabling increased computing power and improved SWaP, has taken the switching speed or clock frequency of the processors from 750 kHz (Intel 4004) to 8.6 GHz (AMD’s FX) [154]. As the processor merits (e.g., the loosely adopted trend of doubling of transistor number on a chip per two years, i.e., Moore’s law) is approaching its current limits, novel processor architectures based on CNTs [149,195,196,197,198,199], or other nanomaterials [146,177] are being explored with tremendous prospects for the next generation computation and communication systems.

SWaP of processor units and related devices are expected to improve by employing the next generation of processors and on-chip integrated electronics [200] based on CNT [47,188,198,199,201,202,203], plasmonics [41,93,145,204,205,206,207], nano-optics, quantum computing [45,131,208], silicon photonics [144,145,209,210,211], optical interconnects (as opposed to metal interconnects) [212], and switches. In cases where the losses in plasmonic systems [213,214,215] may be an issue, all-dielectric resonances are emerging as an alternative approach [137]. 

In exploring new mechanisms and systems for information encoding and transport toward technology alternatives for processors, FPGAs [107], interconnects [115], etc., use of nanotubes [47,149], nanowires [146,223,227,243], ferroelectric capacitors and FETs (FeFETs), and other nanosystems continue to be reported. While some candidate materials of potential use in nanoelectronics and for transferring information, such as CNTs [149,217], plasmonic nanowires [223], and all-dielectric stack optical nanofibers [244], have been discussed above, a more compact presentation of the nanosystems of potential for the development of the next-generation processor and related components is given in Table 1.

Throughout this work, the central theme is how EC could take advantage of nanoscience and the related applications and thus enable the next superior technology. The applications cited seem to be in support of this theme. Our motivation here stems from the natural need for edge devices characterized by mobility and agile computing power at low energy consumption. However, a level of skepticism remains regarding the real progress and impact of EC across this very large range of fields that will be discussed next. Further research is thus warranted to establish the state of EC. 

### 4.1. Carbon Nanotube CPU and Edge Device

A nanosystem of particular interest in nanoelectronics and thus of impact for EC is the carbon nanotube. Further reduction in the size of a large number of transistors fabricated on silicon wafers in the current state of chip manufacturing lead to impractical energy dissipation and transistor failure and thus loss of information. Silicon-based transistors, developed to be increasingly smaller, experience conductivity losses, leading to energy loss by generating heat. Replacing silicon-based elements with CNTs, owing to their more efficient electron transport properties, can lead to less energy requirements. In “How we made the carbon nanotube transistor” [166], C. Dekker provided a brief account on the creation of room-temperature transistors based on a single CNT as the channel material instead of bulk silicon [217,245]. 

CNTs are large aspect-ratio quantum wires [196,216,246,247] with promising electronic, plasmonic [145], thermal, and mechanical properties [188,248], and can be arranged and integrated compactly [47,198,200,249,250,251,252] towards new computer systems [217,253,254,255,256,257,258,259,260,261,262,263,264,265,266,267]. CNTs, as single carbon-atom thick cylindrical current carriers, have micrometers long electronic coherence lengths with electron mobility ~70 times higher than silicon and ~25% higher than other semiconductor materials. With the increasing number of bits of information that has to be processed via charge transport through the transistors, the superior mobility of the CNTs is significant. Following the creation of the first CNT transistor in 1998, logic gates [219] and, eventually, central processors have been demonstrated [268].

CNTs have a set of demonstrated advantageous properties, such as thermal, optical, and electronic properties. For example, mixed CNTs, containing both multi- and single-walled carbon nanotubes (MWCNTs and SWCNTs), have been also proposed as an interconnect for VLSI (very-large-scale integration) circuits [269]. Interestingly, CNT-polymer mixtures have been employed as the dynamical substrate in reservoir computing [270], a recurrent neural networks framework suited for dynamic (temporal) data processing [271]. The measurement of transport properties of individual CNTs [220] has identified both metallic [218,272] and semiconducting [245] characteristics. Similarly, both ballistic and diffusive phonon transport [221] of CNTs have been investigated at room temperature [273,274]. The determination of the electronic scattering mechanisms and quantized energy spectra [275] help exploiting these novel nanomaterials. Further characterization of CNTs, single and bundled (e.g., for allowing larger and faster transport than copper interconnects), as a function of CNT length, contact geometry, wrapping or chirality, and diameter (~0.4 nm–2 nm) will aid the future design [276,277,278].

Single-walled CNTs are cylindrically rolled-up graphene layers with useful electronic band structure and transport properties [279,280]. Many CNTs exhibit direct bandgap. CNTs’ semiconducting bandgap approximately scales inversely with their diameter. With typical energy values of 1 eV for a 1 nm diameter CNTs, they are also of potential use as a quantum light source [216,279]. The general light emission properties of CNTs are via incandescence, electroluminescence, and photoluminescence. The intensity of the emitted light from the excitonic states can be controlled by current modulation (~GHz) and be enhanced via coupling to a cavity [281]. By engineering the photonic density of states via plasmonic or photonic environmental design, e.g., by use of a nanophotonic crystal cavity, it is possible to achieve proper modal volumes, optical quality factors, and Purcell enhancement, and thus a controllable optimum CNT-based on-chip light source [281]. 

Naturally, CNTs are p-type semiconductors, but n-type CNTs can also be achieved, for example, via electron doping (surface charge transfer from an electron donor) [282]. While efforts to incorporate CNTs as the transistor channel or as interconnect materials are ongoing, field-effect transistors (FET) and related development based on CNTs have been demonstrated [188,195,197,198,199,217,246,250,268,283,284,285,286,287,288,289,290,291], including results from IBM [167,292,293]. Very recently, using the CNT FETs, hyperdimensional computing has been experimentally demonstrated [47,149]. 

Various transport properties of CNTs (electrons or phonons in quantized and ballistic conductance) [221,294] may be exploited in a CNT-based transistor [217,268] or device, which is envisioned to outperform (same scale) silicon-based transistors. The appeal of higher speed and lower voltage operation of the CNT-based FETs derives fabrication and experimental efforts to achieve smaller system with recent reports of 5 nm gate length transistors conducting a larger current at 0.4 V than that achieved from the best silicon CMOS transistor (Intel’s 14- and 22-nm Si CMOS FETs [295]) at 0.7 V [296].

Statistical estimates suggest that about 33% of fabricated CNTs exhibit metallic [218,272] rather than semiconducting properties [245,297]. With the current target purity of 0.0001%, various innovative approaches have been proposed to “filter” CNTs. Practical issues related to sorting between metallic and nonmetallic CNTs [298,299,300] for purity in response, nondestructive alignment on a chip [169,291,301], and overcoming CNT-metal junction barrier that limits transistor conductance [302], are being addressed. With improved implementation, multiple CNTs can be used to channel sufficient current in a transistor. To achieve sufficient current per transistor, >125 CNTs/μm has been targeted [297]. Thus, the superior semiconducting properties of CNTs can be exploited in practical devices for faster switching (~10^15^ s) and thus better chip-making materials. 

With a density of states of zero at the Fermi energy, graphene behaves as a zero-gap semiconductor or a metal, which can be inherited by the CNTs. With carbon atom each covalently bonded to the three neighboring carbons via their sp^2^ states in a hexagonal lattice, the electronic band structure of the CNTs has been studied when investigating its transport properties [278,303]. 

The International Technology Roadmap for Semiconductors (ITRS) [304] is calling for “miniaturization of logic transistors by the progress of technology “nodes,” with smaller numbers indicating newer technologies for smaller and faster devices.” The prediction of ITRS for the device gate length is a reduction to 10 nm, with the contact critical dimension reduced to 11 nm, and 4-nm spacer width, to achieve an overall device footprint of 40 nm in the 3-nm technology node. These specifications have recently been met in a CNT transistor scaled to a 40-nanometer footprint [305]. Thus, CNTs will continue to play a major role in the next-generation processors, as well as in many sensors, both of which will enable powerful EC devices. 

### 4.2. The Topological Edge States to Aid Edge Computing

In the ongoing efforts to increase the computing power while achieving an optimum SWaP that meets the demands of EC, work on 2D materials are gaining considerable momentum [306]. The effort is focused on achieving superior electronic transport properties in 2D materials when compared to silicon. With such superior materials, nanoelectronic devices (e.g., FETs) are envisioned to exhibit superior electrostatic control over their transport properties. Recent proposals on developing FETs based on 2D topological insulator ribbons are promising [307]. The transistor switching in topological FETs can be controlled via modulation of scattering since in topological materials [308] electrons can be protected from backscattering allowing the domain to lack resistivity. 

Topological materials [170,172] are emerging as a new class of materials of great potential for novel optical, electronic, and quantum hardware. Such materials possess interesting surface or edge states that may enable high-speed and low-power computing and communication devices. Topological behavior can be exhibited by various electronic, photonic, and atomic systems, where the existence of specific states (e.g., edge states) facilitate electron conductance, photon propagation that is insensitive to defects, disorders, and other disturbances [176]. The link between time-reversal symmetry (and other symmetries) and topology is of great importance in the electronic and photonic response of materials, such as topological insulators, i.e., materials that conduct charge on their surface but insulate in their interior [173,309]. The geometry of the electronic band structures in topological materials exhibits various arrangements that allow for unconventional surface states. Very recently, exploring the high-symmetry points in the Brillouin zone of various materials, nonmagnetic topological electronic materials have been cataloged [170] by calculating their invariant properties. Remarkably, Zhang et al. found that more than 8056 out of 26,688 materials examined are indeed topological, rendering nontrivial topology not exotic and scarce but abundant [170]. Concurrently, Vergniory et al. reported a similar finding by analyzing 26,938 stoichiometric materials (from the inorganic crystal structure database) [172]. Again, remarkably, 3307 topological insulators and 4078 topological semimetals were identified. As a result of their elementary or irreducible band representations (i.e., electronic bands that cannot be decomposed), Vergniory et al. estimated that more than 27% of all materials in nature were topological (out of which an estimated 12% were topological insulators) [172]. 

Consideration of topological properties of material within their electronic or photonic band structures, in analogy with mathematical topology, which links certain conserved quantities with continuous deformations, has recently generated an array of novel materials. The discovery of topological insulators, for example, has led to the demonstration of electron transport without dissipation, even in the presence of disturbances, such as defects and impurities. Similar to the specific topologies in the electronic band structures that support such useful transport properties, topologies in the wavevector-space can be created that supports photonic states, where light propagation can occur on the edges or boundaries without disturbance. For example, in novel materials, such as photonic crystals, and metamaterials, photonic waveguides may be designed to allow light propagation without back-reflection in the presence of imperfections [242]. 

Topological properties of low-dimensional materials are of particular interest since materials, such as graphene and CNTs, are already being explored for use in next-generation processors. For example, single-wall CNTs have been reported to be a topological insulator, due to winding number invariance (determining the number of edge states localized at the tube ends) [310]. With device feature sizes ~7 nm, CNTs are currently considered to be the most practical alternative for a transistor [297]. Use of topological materials to achieve processor size and performance advantages shares similar challenges as CNTs, including large scale fabrication and integration. 

### 4.3. Nanophotonics and Plasmonics to Aid Edge Devices

The utility of light and optical excitations in various nanosystems is of tremendous importance for EC. In general, communication may be regarded as a transfer or exchange of information. Modulation is the essence of communication. Measurable variations in a quantity, such as the electric field amplitude, phase, or polarization, can provide a route to modulation and thus can convey information. Early examples include native American’s messaging by smoke profile modulation. High-frequency modulation of light attributes is essential and can play a particular role for EC, where fast data processing and transfer is needed. Light-by-light modulation, an all-optical scheme, may provide a solution. However, direct light-by-light interaction is too weak, at least within the linear regime. Optical modulators typically rely on weak nonlinear light-matter interactions to accomplish light-by-light modulation. This is where the optical surface excitation of nanoparticles can provide a unique advantage. One approach is to utilize plasmons in communication. How do we implement a surface plasmon-based communication in a potential system/device? The physical implementation of modulation, using plasmon supporting nanostructures, offers a path. However, despite large volumes of investigation, no concrete consensus has been reached as to which physical system is most appropriate for light-by-light modulation. 

Photons can outperform electrons in many aspects of communication and high-speed information networks, including long-distance communication. Additionally, both classical and nonclassical states of light (e.g., coherent and squeezed quantum states) are being explored for the development of novel processors. Furthermore, photons can couple to collective electronic density oscillations, i.e., plasmon excitation and other quasiparticles in solids, and cause a range of fascinating near- and far-field phenomena. Plasmonic devices [205,207,311,312,313,314] are envisioned for several potential applications, including facilitating readout, amplification, modulation, etc., leading to on-chip nearfield devices and achieve operations, such as plasmon-induced transparency [315]. Quantum light (e.g., single-photon) sources [216] and detectors integrated with nanophotonic components provide a basis for developing all-optical processors [43], secure fast communications, and metrology. These features will accelerate the development of EC devices and can play a key role in the emerging quantum networks edge-nodes, or AI hardware platforms [316]. 

Fiber-optics introduced a drastically improved communication by capitalizing on the field propagation properties of fibers and the capability of modulating the fields. To be viable and a superior alternative to silicon chips, new photonic processors [40,41,42,44] must permit a high level of integration [45,317]. However, many optical systems are limited by diffraction, scattering, and losses. Additionally, (light-by-light) modulation, as the essence of information, is challenging, in particular, within the linear regime. However, in the nonlinear regime, optical modulation is possible by exploiting the weak light-matter interactions. Such modulation of light with light [213] can be enhanced or enabled via excitons in quantum dots [228,233], plasmons in nanowires [223,224], and 2D materials [318,319,320]. As a result, metallic, dielectric, and metallo-dielectric composites and multilayer thin films and particles, metasurfaces [239,241] and metamaterials [240], photonic crystals [321], and topological [322,323] and quantum materials [324] are all being actively explored to achieve new photonic functionalities [43,45]. Use of these emerging materials, in conjunction with light-matter interactions and the ensuing electronic, photonic, and phononic excitations (e.g., surface modes, plasmons, polaritons, etc.) [144,145,318,325], is paving the way for the development of compact, addressable, switchable, and optical components and systems [40,41,204,212,326]. An example is the integrated microwave photonics [327], where ultra-small high-bandwidth components, such as electro-optic modulators, frequency synthesizers, and chip signal processors, have been developed. Thanks to the maturity of the semiconductor industry, the integration of photonic components (light sources, detectors, splitters, modulators, polarizers, etc.) in a single microwave photonic processor chip is feasible [40]. Therefore, the development of optical signal processors [40] capable of similar functionality, modularity, and configurability as those of the electronic circuits is possible. The tremendous implication and impact of such communications and information processing capabilities and the perspectives of interoperability and relation with quantum [45] and neuromorphic photonics have been recently reviewed [327].

The potential of photonics to achieve high-speed computing and information processing [40,42] is also noticeable from on-chip devices capable of integrating [44] or solving differential equations [209]. 

While many photonic devices have been recognized to be of potential, to be a viable technology, integrated photonics is envisioned to take advantage of the well-established microelectronics CMOS foundries. Silicon photonics aims to fabricate passive and active photonic circuits on silicon substrates [211]. The very-large-scale integration of electronics and photonics to create dense electronic-photonic systems, on chips, will benefit processors, memory interconnects, ultrahigh bandwidth RF (radio frequency) signal processing, photonic A/D conversion, and other applications in optical interconnects and optical signal processing [328]. Monolithic photonic integration by use of state-of-the-art CMOS foundry infrastructure has been demonstrated to produce large numbers of nanophotonic devices integrated with high-density transistors. Specifically, sharing the same CMOS process mask set, grating-coupled microring-resonator filter banks [211] have been fabricated to achieve wavelength-division-multiplexing [329]. Similarly, fabricating optical waveguides and resonators, high-speed optical modulators and photodetectors, in conjunction with millions of transistors, operations at ten gigabits/s in a single optical bus for wavelength division multiplexing, has been reported [330]. Complete on-chip electro-optic modulators and systems (~5 Gb/s) and interfaces (including infrared detectors and receivers) to improve the processor-to-memory communication energy have been demonstrated for 5 fJ/bit operation at 1.5 dB insertion loss and 8 dB extinction ratio [331].

Emerging nanomaterials and functional low-dimensional systems are particularly appealing as the physical basis for new processors, memory, and interconnect architectures. The VCSEL (vertical-cavity surface-emitting laser) technology has been explored through major initiatives for connectivity in modern data centers [332]. Connecting multiple servers, memory, and computing resources, optical interconnects at terabit/s is an enabling technology for the next-generation data centers [333]. Within integrated photonics and computing architectures, important challenges remain regarding photonic network-on-chip and chip-to-chip architectures [334]. However, significant flexibility is offered by photonics. For example, an information-carrying photon may be converted to an information-carrying plasmon that can then propagate through a quantum plasmonic [335,336] circuit in an optical computer or processor. The clock of a plasmonic chip may be designed based upon the surface plasmon amplification by stimulated emission of radiation (spacer) [337]. 

Undoubtedly, the collective electronic (e.g., plasmonic) effects in nanoparticles and nanostructures of certain materials are of potential importance in information and communication science. When the charge density is perturbed out of equilibrium, coherent electron oscillations can propagate through the material. When quantized, plasmons, the quanta of the density waves, can be created or annihilated, leading to a resonant scattering, absorption, field enhancement, and confinement of potential use in novel optical switches and modulators, which can be employed in integrated photonic devices, in the development of new on-chip or chip-to-chip interconnects, and in achieving computing functionality and implementation of a cellular automata algorithm [338]. Plasmon excitation and transport in CNT and graphene nanoribbons have been explored for their role in conduction in electrical interconnects in the THz range [152]. 

With the current trend of megawatts’ energy consumption by data centers and HPCs, new low power and power-efficient interconnect technologies are needed for exascale computing and beyond. By 2020, the traffic in data centers is projected to be ~15 Zettabytes, i.e., a two-fold increase from 2017, with nearly 77% of the traffic accounting for intra data center needs [339]. Optical, photonic, and plasmonic interconnects are of potential for achieving the needed energy-efficiency and bandwidth-density [340].

Further flexibility offered by photonics is in crystals where photon propagation is governed by the naturally occurring periodic structure. Of use is propagation that can be manipulated or prohibited over some spectral bands, that is feasible in materials possessing artificial periodic structures. Such photonic bandgap materials are useful for optical processors (switches, modulators, etc.) similar to semiconducting materials in silicon-based electronics. In many cases, numerical and computational techniques, such as the finite difference time domain (FDTD) and time or frequency domain finite element (FE) methods, can be employed in the understanding (forward problem) and design (inverse problem) of photonic materials toward specific processing needs. Since photons are bosons, they do not readily interact, making the photonic device calculations easier, typically requiring the solutions of the photon wave equation (Maxwell’s equations). Compared to the determination of the response of the electronic systems, requiring the many-body interaction (electron correlation) or the solution of the many-particle Schrödinger equation, such photonic advantages are however offset by the difficulties in localization and confining photons, making device miniaturization and integration nontrivial. 

Most metal-based nanostructures that support collective electronic excitation are dissipative and generate heat (e.g., nonradiative decay of plasmons [341,342]), and thus may ultimately not be suitable for tight integration. Such challenges may be circumvented by the use of all-dielectric devices, which are promising candidates for the control and confinement of light [135,137]. 

### 4.4. Quantum Processor and Computing to Aid Edge Devices

Effects, such as scattering, and losses sustained by electrons confined to smaller dimensions can limit their use as information carriers or in signal transduction. However, the fundamental physical phenomena that place limitations upon the further increase of the density of the conventional information processing materials also offer new opportunities for drastically breaking the conventional computing records [208]. Capitalizing upon quantum effects, such as the distinct polarization states of a photon or the spin states of an atomic or subatomic system, quantum computers [131] are envisioned to achieve operation on data with merits beyond the reach of any classical computer (the so-called quantum supremacy). Extensive research is ongoing to stably represent and manipulate information carried by a quantum system. Unlike the use of binary digits (bits) in classical computing, in a quantum computing platform, information is encoded using quantum bits (qubits); for example, the two orthogonal polarization states of photons or the states of a two-level quantum system and the superpositions of those states. Changes in the state or operations on the qubits lead to information processing. 

Quantum dots made of composite metal, dielectric, or semiconductor materials are of potential as physical systems for hosting qubits. Being essentially 0D materials, their nanoscale dimensions allow arrangements of a large number of nodes on a surface with sufficient spatial separation to preserve the integrity of the qubits in a quantum processor. 

Spin, as an intrinsic property of particles and measured to assume different quantized states for bosons (integer values) and fermions (half-integer values), has been identified as an information carrier for computing. For example, the spin (and charge) states of a semiconducting quantum dot allow it to be an ideal nanosystem for quantum computing. Further information on scientific advantages can be gleaned off by fermions and bosons obeying Fermi–Dirac (preserving Pauli exclusion) and Bose–Einstein (not preserving Pauli exclusion) statistics, respectively. Bose–Einstein condensate, using cold atoms (e.g., the alkali metal rubidium) that can be trapped or can trap an ion, shows fascinating many-body behavior of potential use in quantum devices [343]. 

In addition to photons as the information-carrying particles, dopants in silicon [344] or defects in diamond [345] are also being explored as potential atomic-scale qubits with stable coherence time and operation at room temperature. In diamond, a vacancy in the atomic structure can possess useful spin properties. The nitrogen-vacancy (NV) centers exhibit useful electron-spin coherence and relaxation times [237]. Similarly, qubits may be obtained from 2D materials, such as molybdenum disulfide [177] or graphene [206,319,320], which can host single donors with charge and spin states that can be controlled.

The predicted impact of quantum information science (e.g., quantum computing [45] and information processing), AI, machine learning, and their convergence [346] on EC devices will be hard to overestimate [347]. To realize quantum computing [45], quantum internet [347], and by extension quantum edge devices, a central topic is the identification of qubits that possess long coherence times, that is, sufficient times under which the information-carrying system stays in a superposition state to allow the transmission of information. Insufficient coherence times lead to the loss of quantum information and errors. Quantum gates operate on qubits, and thus the coherence time should be much longer than the time the gate needs to complete its operation [345]. To achieve a processor capable of controlling many qubits, the platform has to be scalable, and any errors due to noise and loss of coherence must be corrected (quantum error correction and fault-tolerant quantum computation) [348]. To benefit from quantum computing, in addition to the quantum hardware, development of quantum algorithms is also necessary. 

For an object in an arbitrary unknown quantum state, one cannot precisely measure or perfectly replicate that state. However, the state can be teleported to another object, that is, transfer of the state without the physical transfer of the object. These and similar effects are being currently considered for long-distance communication [349,350]. The EC device communication could greatly benefit from such quantum effects, which have been demonstrated using optical fibers and terrestrial free-space channels over distances of ~100 km, and free space channels over distances of ~1400 km [349]. 

Quantum edge devices may be readily envisioned to incorporate quantum sensors that generate data to be processed by quantum processors and algorithms and communicated through quantum internet [347,349]. Further prospects of EC may be anticipated from the emerging distributed quantum sensing [351], where quantum correlations are exploited between multiple sensors to achieve superior measurement of unknown parameters overcoming the limits of unentangled sensors. The emergence of the powerful capabilities of EC and quantum sensing, distributed or not, warrants an investigation of their specific and unique synergy. An important concern regarding the expansion of EC devices is security, for which quantum effects may provide powerful solutions [350]. Quantum key distribution is one such potential solution, where unconditional communication security between distant parties can be guaranteed by working with photons in quantum superposition states [350]. Additionally, cyber-physical systems are evolving into a state where processing occurs at the distributed sensing layer and not in a centralized manner [352]. EC will need new hardware platforms consistent with increased computing performance. Thus, distributed computing can bring physical sensors to a whole new potential for quality and operational capabilities. 

An important application area for EC is cellular technology. Satellites provide services by accessing ground computing centers to transmit data. This process consumes a large amount of bandwidth and generates a delay. To improve resource utilization, computing power, and delay, the ground 5G mobile communications network employs EC. The mobile EC (MEC) distributes part of the computing resources to the edge of the network, thus saving bandwidth and reducing delay. For further improvements, a 5G satellite EC framework has been proposed [353] in addition to existing EC for terrestrial 5G communication. In addition to EC’s potential to overcome the problems anticipated for cloud computing, further capabilities may be created by exploiting quantum effects that drastically boost satellite communication, as evident from a recent report, where teleportation of single-photon qubits was employed for ground-to-satellite communication [349]. Another impressive example is the recent report on using quantum key distribution to achieve secure communication from the satellite to the ground [350]. Thus, global quantum networks may become a reality in the near future. As such, despite the early stage of quantum technologies, efforts are underway to investigate and develop quantum networks edge-nodes. Such nodes are defined to be network locations capable of hosting quantum subsystems, such as photon detectors, transmitters and receivers, quantum buffers and memories, etc. These nodes are needed for connecting quantum information system applications (e.g., a quantum sensor) to the optical quantum networks. Quantum networks edge-nodes are then considered to provide peer-to-peer services at the quantum applications layer, i.e., a distributed application architecture. Through such an architecture, tasks and workloads are partitioned between equally privileged participants (peers) in the quantum application. An example of such peer-to-peer service is the ability to convert stationary qubits (e.g., generated by a trapped ion or other quantum applications) into flying qubits, which are suitable for transmission via optical fiber communication systems. Other service examples include transmission of flying qubits between specified locations. While the potential advantages of quantum processing for EC require further maturation of the quantum information science, the concept of a quantum edge device may stimulate further research.

### 4.5. Neuromorphic Computing and Edge Devices

The advances in the discovery and development of new HPC processors will undoubtedly impact the deployment of countless EC and IoT devices that are being envisioned. Since EC envisions to bring intelligence and connectivity to virtually all aspects of sensing, fast processing and communication of the large-scale (per sensing element) data generated is critical [354]. This is not unlike the amount of data and computing power of the sensory organs and brain of a human (~1 GB/s data from the eyes alone; and 10^16^ brain operations/s [355,356], and ~20 W power consumption [357]) to produce a decision. In the traditional computing realm, the memory and processing unit are separated, limiting data communication rate (the so-called von Neumann limit). Neuromorphic computing [358] is touted as a potential approach to address the inherent limitations of conventional silicon technology. Biological systems exhibit complex dynamics and responses that are being increasingly exploited in many fields, including sensing and computing [359,360,361], for example, as described in “neuromorphic engineering” [356], where a brief account is given on some of the early developments of brain-like technologies and neural circuits. Neuromorphic or brain-inspired computing, unlike the serial processing of traditional digital computers, is envisioned to achieve massive parallel analog computing at high speed and low power [362]. To be comparable to the brain, neuromorphic hardware requires ~10^11^ neurons [363] and thus needs to be extremely energy-efficient. An increasing number of studies are emerging toward achieving the basic elements needed to build neuromorphic devices [357,364,365,366,367,368,369], including the recent work on “evolvable organic electrochemical transistor”, which was reported to mimic the biological synapse [148]. Remarkably, similar to biological synapses, which establish, evolve, and operate, the devised transistor channel, formed by an electropolymerized conducting polymer, the first synaptic device was produced that generated new synapses within its working environment [148]. 

In the neural system, the synapse functions as an active memory unit, enabling learning and memory (with an energy consumption of ~1–100 fJ per synaptic event [370]. A logical approach to implementing neuromorphic processors is the emulation of a synapse [354], which requires a compact and scalable physical basis if it is to be integrated into a three-dimensional architecture [365]. For example, intended to operate analogously to a synapse between neurons, a memristor [357,371] can alter its resistance dynamically depending on its history and thus can possess multilevel accessible conductance states, a useful feature for neuromorphic systems [372,373]. Memristive devices have also been used in sparse coding and dimension lowering, envisioned to aid neuromorphic computing [355,374]. Similarly, nanoscale spintronic oscillators, exhibiting nonlinearity, memory, and stability, have been experimented with to emulate collections of neurons [375]. Other recent developments, related to mimicking a synapse or synaptic behavior, include an electrochemical neuromorphic organic device made of inexpensive plastic material [376], neuromorphic circuits to emulate a sense of touch [377], memristive response using ionic effects in MoS_2_ [378], quantum effects in superconducting circuits [366], photonic integrated-circuit synapse [354], quantum dots-based photonic synapse [370], oxide-based photonic synapse [379], single flux quantum circuits [380], and use of aligned CNT transistors [381]. 

In memristors, conductance can be modulated continuously by applying a voltage, a feature that can be used to naturally implement analog vector and matrix multiplication. How precisely the modulation occurs depends on the number of conductance states. Memristors, allowing analog computing, are particularly useful for EC and IoT as faster and more energy-efficient computing can be achieved when compared to conventional digital computing [382]. An example is a vector-matrix multiplication, which constitutes a core computing task, e.g., in signal processing and deep neural networks, both of which are fundamental for EC. Memristor crossbars, providing reconfigurable non-volatile resistance states, can be used for efficient analog signal and image processing as an alternative processing method for EC and IoT. Memristors could thus eliminate the speed and energy efficiency bottleneck. Using hafnium oxide memristors on top of metal-oxide-semiconductor transistors, signal processing, image compression, and convolutional filtering have been demonstrated [383]. Other exploratory investigations using memristor arrays include solving classic control problems associated with reinforcement learning algorithms that use deep neural networks [384]. 

In the quest for a competitive neuromorphic CPU, the experimental system of IBM stands out. The TrueNorth with a 5.4 × 10^9^ transistors chip was reported in 2014 [385]. It featured 1 × 10^6^ spiking neurons and 256 × 10^6^ synapses but a power density of only 20 mW/cm^2^, compared to tens of W of typical CPUs. A comparison of power densities and clock frequencies of processors with those of the brain has been reported by Merolla et al. [385]. Industry research efforts in developing neuromorphic processors are also producing novel results. The 60-mm^2^ chip Loihi is a neuromorphic many-core processor fabricated on Intel’s 14-nm process [386,387]. It implements spiking neural networks with on-chip learning and has been shown to outperform other approaches in solving the LASSO (Least Absolute Shrinkage and Selection Operator) optimization problem [386,387].

### 4.6. Discussions

Having briefly surveyed the rapidly growing number of EC-related reports (see Figure 1) and considering the state of the technologies discussed above, the true contribution of EC to these fields appear modest or hard to assess otherwise. Many of the fields noted are oblivious of EC. To date, the reported EC-related studies appear to be largely extensions of cloud-based scenarios, and considerations and are only weakly connected to what may be perceived as uniquely EC. On the one hand, the technologies above appear to offer or be of potential to offer hardware components and ingredients towards building the core systems of an edge device (e.g., low power high-performance processors). On the other hand, there are a number of algorithms and software efforts that aim to distinguish EC as a new computing paradigm (e.g., EC simulators [116]). Without catalyzed interaction between these two domains, progress may remain modest. While it may be challenging to sketch a road map for achieving the EC vision with respect to nanosystems, for these seemingly disparate fields to cooperatively achieve the EC vision, dissemination and exchange of the core characteristics of EC need to occur across these disciplines. In a sense, one may consider the traditional state of biology versus the emerging state of physical sciences biology, where increasingly models, theories, and experiments from mathematics, physics, and engineering are transforming our understanding and use of biological systems. In turn, biology stimulates and poses new challenges to physical and mathematical modeling and theories of physical systems. The EC could benefit from a similar convergence. For example, one may consider a molecular biologist as an EC investigator addressing questions, such as the development of a local information storing or computing protein to serve the processing needs of a local biomolecular sensor. Such problem statement within biology may be of potential to break the barrier between the fields.

## 5. Conclusions

While efforts to formulate uniquely EC-use cases are underway, the presented review sketches the overall state of the field. Whereas electronics may have been a major precursor of EC, nanoelectronics is here emphasized as essential for the evolution of EC. In an all-inclusive EC value-chain and ecosystem, nanoscience is an essential stakeholder. From the perspective of novel systems of potential for EC-specialized applications, discerning the technological challenges in achieving faster, smaller, and more energy-efficient processors and components, including interconnects, storage, communication, and software, helps to define the broader scope of the EC as a field. Clearly, despite the growing number of reports that are sharpening the boundaries of the EC field, the cross-disciplinary nature of the field must also be considered. This is not unlike the cross-disciplinary field of nanoscience, which provides the potential hardware and implementation solutions for EC, as described in the case of CNT-based transistors for post-Moore needs. However, in turn, emerging unique EC-use cases also provide new challenges for nanoscience. For example, from the presented discussion on CNT synaptic transistors for neuromorphic computing, it may be surmised that nanosystems and EC may amalgamize to become an inseparable entity, where device and function interact dynamically. Measuring the global needs for better and new sensors, it can readily be concluded that increasing the computing power and the intelligence of the sensors is only a natural evolution of the related industries. The majority of EC devices are expected to be fast, compact, low power, and resilient to becoming compromised (hardware and software). The processor unit that could give the EC sensors and devices the needed intelligence to provide an “edge” advantage needs also meet these same criteria. Remarkably, EC appears to impact a plethora of scientific and technological fields as it overcomes many challenges, such as the potential risk for increased hacking vector and licensing costs. Clearly, important challenges remain to be addressed with respect to security, software upgrade, and others related to the touted EC advantages. From the brief review presented, it may be concluded that the role of nanomaterials and nanosystems in overcoming these obstacles takes the center stage. Not only the hardware that accommodates the computing power, intelligence, and communication capacity of the EC devices but also the signal generating sensors and controlling actuators benefit from the extraordinary properties of the reviewed materials. With the rising fusion of the IoT devices globally, EC could help to unburden the resulting colossal computing and communication loads. In turn, the evolution of EC devices and the synergy among the sensing, computing, and AI components are expected to enable new scientific and technological capabilities. As the EC device dimensions are pushed to smaller scales to achieve higher density integration, the boundaries of nanosystems and EC further merge. It may be argued that the state of the nanosystems-EC field is only at its infancy with such perspectives as collective and holistic responses from high connectivity, synchronized, and real-time EC devices may be tamed to provide new horizons of information technological capabilities. Mathematical problems, for the spatial distribution of edge devices, or the rate of computing and data exchange, etc. may be formulated, to better understand the underlying complexity, and thus emergence. EC will reach its climax as novel nanosystems that switch fast, dissipate less energy per switching, implement functions, transport information-carrying signals fast and with little dissipation per unit length are discovered and harnessed. Currently, materials that exhibit topological and quantum behavior, metamaterials, and nanoparticles and nanowires that can be integrated are under consideration. With evolving nanosystems, one may envision molecular systems, e.g., proteins and DNAs, as EC devices. With sensing at the atomic and molecular levels, nanoscale communication, molecular processing, and nano-EC devices may pave the way to nano-IoT. Molecular networks of billions of sensors already occur in biological systems, and this may be mimicked by nano-EC devices. Can the human body be regarded as a centralized cloud-based system? How would an EC-augmented human be envisioned? In the tradition of cloud-computing, nanosystems and EC will stimulate future applications with far-reaching impact. 

## Figures and Tables

**Table 1:** Novel nanomaterials and nanostructures of importance in the research and development of the next-generation computing systems.

Nanosystem	Typical Excitation	Application	References
Carbon nanotubes	Electron-hole transport	Transistor channel, cooling, vias, connectors	[216,217,218,219,220,221]
Nanowires and nanoantenna	Plasmons	Interconnect, connector, qubit	[146,207,222,223,224,225,226,227]
Quantum dots (doped, undoped Si, GaAs, etc.)	Excitons	Qubit	[226,228,229,230,231,232,233,234,235]
Silicon photonics components	Donor, electron, hole charge and spin states	Transistor material, qubits, quantum computing	[176,210,211]
Nanophotonics components	Photons, polaritons, plasmons	Transistor material, qubits, quantum computing	[204,216,231,233]
Organic compounds	Charge	Transistor material	[147]
Nitrogen vacancy	Spin states	Qubits, quantum computing	[205,236,237]
Trapped ions		Qubits, quantum computing	[131,238]
Nano- and micro-electromechanical systems (NEMS and MEMS)	Phonons	Readout	[193]
Superconductors	Electron	Qubits, quantum computing	[222,226]
Metamaterials and metasurfaces	Photons, phonons, plasmons	Transistor material, frequency conversion	[94,239,240,241]
Topological materials	Photons, phonons, plasmons, electrons	Interconnect, connector, qubit, transistor material	[170,171,172,173,174,176,222,242]
Metal-oxide-semiconductor and -metal tunnel junctions	Electron, plasmon, photon	Transistor material	[232,234]
Multilayers	Bloch surface waves	Interconnect	[94,135]
